# Mapping QTLs for drought tolerance in a SEA 5 x AND 277 common bean cross with SSRs and SNP markers

**DOI:** 10.1590/1678-4685-GMB-2016-0222

**Published:** 2017-10-23

**Authors:** Boris Briñez, Juliana Morini Küpper Cardoso Perseguini, Juliana Santa Rosa, Denis Bassi, João Guilherme Ribeiro Gonçalves, Caléo Almeida, Jean Fausto de Carvalho Paulino, Matthew Ward Blair, Alisson Fernando Chioratto, Sérgio Augusto Morais Carbonell, Paula Arielle Mendes Ribeiro Valdisser, Rosana Pereira Vianello, Luciana Lasry Benchimol-Reis

**Affiliations:** 1 Centro de Recursos Genéticos Vegetais, Instituto Agronômico (IAC), Campinas, SP, Brazil; 2 Ciências Biológicas, Universidade Tecnológica Federal do Paraná (UTFPR), Dois Vizinhos, PR, Brazil; 3 Centro de Grãos e Fibras, Instituto Agronômico (IAC), Campinas, SP, Brazil; 4 Departamento de Agronomia, Universidade Estadual de Maringá (UEM), Maringá, PR, Brazil; 5 Department of Agriculture and Natural Sciences, Tennessee State University, Nashville, TN, USA; 6 Bean Program, Centro Nacional de Pesquisas Arroz e Feijão, Goiânia, GO, Brazil

**Keywords:** abiotic stress, interpopulation gene-pool, molecular markers, QTL mapping, water deficit

## Abstract

The common bean is characterized by high sensitivity to drought and low productivity. Breeding for drought resistance in this species involves genes of different genetic groups. In this work, we used a SEA 5 x AND 277 cross to map quantitative trait loci associated with drought tolerance in order to assess the factors that determine the magnitude of drought response in common beans. A total of 438 polymorphic markers were used to genotype the F8 mapping population. Phenotyping was done in two greenhouses, one used to simulate drought and the other to simulate irrigated conditions. Fourteen traits associated with drought tolerance were measured to identify the quantitative trait loci (QTLs). The map was constructed with 331 markers that covered all 11 chromosomes and had a total length of 1515 cM. Twenty-two QTLs were discovered for chlorophyll, leaf and stem fresh biomass, leaf biomass dry weight, leaf temperature, number of pods per plant, number of seeds per plant, seed weight, days to flowering, dry pod weight and total yield under well-watered and drought (stress) conditions. All the QTLs detected under drought conditions showed positive effects of the SEA 5 allele. This study provides a better understanding of the genetic inheritance of drought tolerance in common bean.

## Introduction

The common bean (*Phaseolus vulgaris* L.) is an annual grain legume crop with important human consumption worldwide (Broughton *et al.*, 2003). Drought stress is a serious agronomic problem that contributes to severe yield losses worldwide (Sabadin *et al.*, 2012) and affects 60% of bean production, especially in Africa where this effect is particularly severe (Asfaw *et al.*, 2013). Important bean producing areas that already suffer frequent droughts, such as Mexico, Central America, southern Africa and northeastern Brazil, are likely to receive even less average rainfall in the future because of climate change (Beebe *et al.*, 2011).

A broad understanding of the physiology of drought response is key to identifying useful selection criteria in addition to yield *per se*. The optimal plant response for dealing with moisture deficit will vary depending upon the pattern of drought (Cortés *et al.*, 2013). Four patterns of drought have been defined: late initiation of rains, early cessation of rains or terminal drought, intermittent drought, or low rainfall throughout the season (Levitt, 1972).

Common beans of the Durango race germplasm (prostrate bush types with medium-sized seeds from the dry northern highlands of Mexico) reportedly possess the highest levels of drought resistance and have been used to develop drought resistant bean cultivars in the Middle American gene pool (Singh *et al.*, 2001; Singh, 2007). According to Mukeshimana *et al.* (2014), combining the germplasm of the races Durango and Mesoamerica (small-seeded types, mostly bush habits, from lowland Central America and Mexico; Singh *et al.*, 1991) has provided a consistent source of improved drought resistance for tropical environments. Singh *et al.* (2001) described the SEA 5 line as a drought tolerant cultivar derived from interracial crosses between the Mesoamerican and Durango races; one of the parents originating the SEA 5 line was the cultivar BAT 477.

Genetic and physiological mechanisms related to the responses of plants to water stress are important for the selection of more drought-tolerant plants. In general, drought resistance mechanisms include drought escape, drought avoidance and drought tolerance (Levitt, 1972). Drought escape allows plants to accelerate their cell cycle with early flowering and maturity, and rapidly relocates metabolites to seed production and away from leaves and shoots. Drought avoidance is the ability to maintain high tissue water potential through increased root depth, a reduction in hydraulic conductance, radiation absorption reduction in leaves, a reduction in water-loss area, reduced absorption of radiation by leaf movement, and reduced evaporation surface (leaf area). Drought tolerance is the ability of plants to resist the stress by adjusting cell osmosis, plasticity and size (Levitt, 1972).

Many traits influence tolerance to drought stress, including rooting pattern, the ability to partition a greater proportion of carbohydrates to seeds under stress, the capacity to set pods and fill seeds under stress, reduced stomatal conductance and leaf area, and the ability to maintain turgor through osmotic adjustment (Singh, 2007).

Breeding for drought tolerance is complex because of the number of traits involved, quantitative inheritance and environmental influence (Mir *et al.*, 2012). A large amount of data has contributed to our understanding of the impact of drought on the common bean (Asfaw and Blair, 2012; Blair *et al.*, 2012; Mukeshimana *et al.*, 2014). However, the identification of major-effect QTLs with stable expression across different stress environments is needed to facilitate marker assisted selection (MAS) for drought tolerance in the common bean.

Molecular markers are powerful tools for analyzing the genetic control of complex traits such as drought tolerance (Mir *et al.*, 2012). Asfaw and Blair (2012) used random amplified polymorphic DNA, amplified fragment length polymorphism and simple sequence repeats markers (SSRs) to map a Mesoamerican intra-gene pool cross of drought-susceptible DOR364 and drought-tolerant BAT 477, and detected a yield QTL on Pv08 and a stem carbohydrate QTL on Pv05.

Diversity analysis using intron-based SNPs revealed different patterns of diversity compared to that reported by Blair *et al.* (2009a,b) using SSRs. Mukeshimana *et al.* (2014) identified 14 QTLs for performance under drought in an inter-gene pool recombinant inbred line (RIL) population from a cross of the drought-tolerant line SEA 5 and CAL 96 cultivar; QTLs associated with yield components overlapped, especially on Pv03, Pv07, and Pv09. Villordo-Pineda *et al.* (2016) observed 83 SNPs that were significantly associated with flowering time, physiological maturity, reproductive period, seed and total biomass, reuse index, seed yield, weight of 100 seeds, and harvest index in three cultivation cycles.

The goal of this study was to identify QTLs associated with physiological and yield components under drought and irrigation conditions based on an anchored linkage map obtained from a RIL population derived from a contrasting inter-gene pool cross between drought-tolerant (SEA 5 – Mesoamerican gene pool) and drought-susceptible (AND 277 – Andean gene pool) parents.

## Material and Methods

### Plant material

The population used in this study was a set of 107 recombinant inbred lines (RILs) from the cross SEA 5 x AND 277 created at the International Center for Tropical Agriculture (CIAT, Cali, Colombia). The population was propagated until the F_8_ generation using the single seed descent (SSD) method. The drought-tolerant dry bean line SEA 5 was also developed at CIAT (Singh *et al.*, 2001; Terán and Singh, 2002) and is considered superior to BAT 477 (Pérez-Vega *et al.*, 2011). SEA 5 was developed from the interracial double-cross population TR 7790 = BAT 477/‘San Cristobal 83’//‘Guanajuato 31’/‘Rio Tibagi’. BAT 477 is a cream-colored, small-seeded (< 25 g/100 seeds) breeding line developed at CIAT; BAT 477 has an indeterminate prostrate Type III growth habit and is highly tolerant to charcoal root rot [caused by*Macrophomina phaseolina*(Tassi) Goid]. San Cristobal 83 is a red mottled, small-seeded landrace with a Type III growth habit from the Dominican Republic. Guanajuato 31 is a beige-colored, medium-seeded (25-40 g/100 seeds) landrace of Type III growth habit from the semi-arid central highlands of Mexico. This line has high yields, a high harvest index and is resistant to anthracnose [caused by *Colletotrichum lindemuthianum* (Sacc. & Magn.) Lams.-Scrib.]. Crosses involving Guanajuato 31 indicate that it possesses resistance genes to *C. lindemuthianum* races 6, 31, 38, 39 and 357 (Rodríguez-Soárez *et al.*, 2007) and to race 83 (Alzate-Marin *et al.*, 2009). Rio Tibagi has small black seeds and an indeterminate upright Type II growth habit; this is a popular cultivar in central and southern Brazil. BAT 477, San Cristobal 83 and Rio Tibagi belong to the Mesoamerica race, and Guanajuato 31 belongs to the Durango race. All four genotypes have some level of tolerance to drought, although Rio Tibagi has been classified as susceptible (Singh, 1995). AND 277 belongs to the Nueva Granada race and is of the Andean genepool (Blair *et al.*, 2009a). This advanced line was derived from the complex cross [Cargabello x (Pompadour Checa x Línea 17) x (Línea 17 x Red Kloud)] and gamete selection. AND 277 is known to carry the *Co-1* ^*4*^ (Arruda *et al.*, 2008; Alzate-Marin *et al.*, 2003) and *Phg-1* (Carvalho *et al.*, 1998) genes that confer resistance to anthracnose (*Colletotrichum lindemuthianum*) and angular leaf spot (*Pseudocercospora griseola*) diseases, respectively, but is susceptible to drought.

### Phenotyping for drought tolerance

The experiment was done from January to April 2012 at the Agronomic Institute (IAC, Campinas, SP, Brazil), located at 22°52′40″ latitude south and 47°04′72″ longitude west and an altitude of 685 m. Two greenhouses were set up for the experiment. The first one (water stress) was covered with shade cloth as a sun screen and polyethylene plastic to prevent the entry of water during the experiment, while the second one (well water) was covered only with shade cloth.

Each greenhouse was filled with 428 plastic pots linked to an individual irrigation system that allowed control of the amount of incoming water and the drought stress generated in the experiment. The pots were filled with 12 kg of a soil, manure and sand mixture (in a 3:1:1 ratio). Since pot size can affect plant growth and performance (Pieruschka and Poorter, 2012)**,** we used pots with a soil capacity of 12 kg to minimize the influence of pot size. The soil was adjusted to a neutral pH with lime and watered before filling the pots. The experimental design consisted of completely randomized blocks with four replicates. After one week of letting the soil settle, the pots were fertilized using chemically-formulated fertilizer (NPK 8-18-16) that was applied directly to the soil. The total amount of each nutrient was equivalent to 1.5 g of N, 6.0 g of P_2_O_5_ and 3.5 g of K_2_O per pot.

Soil moisture levels in the pots were measured with 30 watermark sensors (granular matrix sensors) that were randomly installed at soil depths of 20 cm in control and drought stress pots. Before planting, the seeds were rinsed for 1 min in 5% (v/v) NaClO, washed in distilled water and germinated in a Biological Oxygen Demand (BOD) incubator for 72 h at 25 °C. Three seeds of each genotype were planted per pot. During the growing season, irrigations were provided twice a day for a total volume of 400 mL. Every two days, soil water tension was measured, along with the leaf temperature of the plants in the pots containing the sensors, as well as the ambient temperature and relative humidity of each environment. Days to flowering were recorded every day and the mean flowering date was calculated for each genotype.

After 20 days of water deficit, one plant of each genotype and each replication was collected for phenotypic analysis; the others were allowed to grow until the end of the crop cycle to measure yield. At this point, leaf temperature was measured with an infrared thermograph (Telatemp model AG-42D, Telatemp, CA, USA), after which the plants were cut at the soil surface and separated into leaves and stems. The chlorophyll present in the leaves was measured with a non-destructive, hand-held SPAD-502 chlorophyll meter (Minolta Camera Co., Ltd., Japan). Leaf area was determined using a leaf area meter (LICOR model LI-3000). For these same plants, the fresh stem and leaf weights were measured using an analytical balance (BEL Engineering, Milan, Italy) to determine biomass partitioning. Plant parts were placed in separate paper bags and dried in an oven at 60 °C for 48 h after which the stem and leaf biomass dry weights were determined.

The physiological and morphological responses to drought were measured 33 days after planting (DAP), when the water-stressed greenhouse plants reached a mean value of 160 kPa of soil water potential. After the physiological and morphological evaluations, the remaining plants were evaluated at physiological maturity to determine their productivity. In general, the plants were harvested after approximately three months, at which point the number of pods at harvest, number of seeds per plant and number of seeds per pod were determined. In addition, yield and total seed weight and dry pod weight were estimated. The drought intensity index (DII) was calculated as $1-\frac{Xds}{Xns}$, where Xds and Xns are the mean seed yield of all genotypes under drought stress (ds) and no stress (ns) treatments.

### DNA extraction and genotyping

DNA was extracted from 300 mg of powdered lyophilized young leaves from the parents and all the RILs by the CTAB method. DNA concentration was measured in a NanoDrop 2000 (Thermo Scientific) and diluted in Tris-EDTA (TE) buffer (10 mM Tris-HCl, 1 mM EDTA, pH 8.0) to a final concentration of 50 ng/μL and stored at 4 °C.

### Microsatellite amplification and analysis

For microsatellite screening, 594 SSRs were tested for polymorphisms among the SEA 5 and AND 277 lines. These SSRs were previously published by Blair *et al.* (2006, 2008, 2009a), Benchimol *et al.* (2007), Hanai *et al.* (2007) and Campos *et al.* (2011). The amplification reactions included 30 ng of DNA, 1 U of *Taq* DNA polymerase, 1.5 mM MgCl_2_, 0.15 mM of each dNTP, 0.8 pmol/mL of each primer (forward and reverse), 10 mM Tris-HCl and 50 mM KCl in a final reaction volume of 15 μL. The following conditions were used for amplification: 1 min at 94 °C, 30 cycles of 1 min at 94 °C, 1 min at the specific annealing temperature for each SSR, and 1 min at 72 °C, with a final extension of 5 min at 72 °C. The PCR products were visualized on a 3% agarose gel and stained with 1X GelRed (Biotium, Inc. Hayward, CA, USA). After checking the PCR amplification products, they were separated in a 6% denaturing polyacrylamide gel and visualized using silver staining. Molecular mass standards (10-bp and 100-bp ladders; Invitrogen) were included in the runs.

### Single nucleotide polymorphism (SNP) analysis

Genotyping for the 384 SNPs was done using the Vera Code^®^ BeadXpress platform (Illumina) at the Biotechnology Laboratory of Embrapa Arroz e Feijão (Goiania, GO, Brazil). A set of 384 SNP markers, validated through Prelim file (https://icom.illumina.com/Custom/UploadOpaPrelim/) previously identified for *P. vulgaris* (Müller *et al.*, 2015) and derivatives of polymorphism between the lines BAT 477 of Mesoamerican origin and Jalo EEP558 of Andean origin were selected to compose the Oligo Pool Assay (OPA) SNP markers.

For the SNP detection procedure on the BeadXpress platform, three oligonucleotides were used, two allele-specific (ASO) primers for each of the variations of the same specific SNP locus and a third primer (LSO) binding to the 3’ region fragment DNA containing the SNP target. After hybridization, the procedure consisted of extending the regions between the ASO and LSO, followed by melting from a ligase enzyme, thus forming a single allele-specific fragment. This fragment was subsequently amplified using the enzyme Titanium *Taq* DNA polymerase (Clontech Laboratories Inc., Palo Alto, CA, USA) and primers complementary to the ASO region were labeled with Cy3 and Cy5 fluorescence.

Finally, the PCR products were hybridized with the complementary region of LSO strings present on the surface of the holographic beads. SNP genotyping was done using the program Genome Studio version 1.8.4, (Illumina, USA), with call rate values ranging from 0.80 to 0.90 and ≥ 0.26 for GenTrain grouping of SNPs. Clustering (grouping) to call alleles for each SNP was done *a priori* in an automated manner based on the intensity of the signals from Cy3 and Cy5. These signals were grouped into three classes of genotypes representing homozygous (AA and BB) and heterozygous (AB) alleles. For data analysis, the groups were adjusted individually and manually by determining the best clusters based on the parental profile.

### Linkage mapping and QTL identification

Segregation analyses the SNP and SSR scoring of the 105 recombinant inbred lines and the SEA 5 and AND 277 parents were done using the chi-square test (*X* ^2^), assuming 1:1 segregation ratios, with Bonferroni corrections. The genetic map was constructed with OneMap software version 2.0-1 (Margarido *et al.*, 2007) using the multipoint approaches and hidden Markov models for analysis in the RIL population. Briefly, after identifying the redundant markers and segregation distortion, the recombination fractions were estimated between each pair of markers using the two-point function. The markers were then assigned to the chromosomes using a LOD threshold of 3.0 and maximum genetic distance of 37.5 cM in conjunction with the Kosambi (1944) map function and the make.seq function. For the remaining markers, the try.seq function was used.

The positioning of the markers was refined using make.seq and map functions consecutively. To help decide on the position of each marker inserted in a specific linkage group, the rf.graph.table and draw.try=TRUE function were used to display the heat map. The nomenclature of the chromosomes and physical positions were identified by comparisons through sequence similarity analysis using BLASTN against the *P. vulgaris* G19833 Andean genome (https://phytozome.jgi.doe.gov/pz/portal.html#!info?alias=Org_Pvulgaris) and the integrated genetic map for the common bean based on microsatellite mapping described by Blair *et al.* (2011) and Campos *et al.* (2011).

Normality of the phenotypic data of the least square means (LSMeans) distribution was assessed based on the skewness, kurtosis and Shapiro-Wilk values. The Box-Cox transformation was applied and the appropriate model for normalizing the data of each trait was selected using the lambda (λ) parameter (Osborne, 2010).

Quantitative trait loci identification was done using QTL Cartographer v. 1.17 (Basten *et al.*, 2005) with composite interval mapping (CIM) analysis. The likelihood ratio test (LRT) was used to check for the presence of QTL at 1 cM walkspeed and 10 cM window size. The coefficient of determination was calculated for each interval separately (R^2^) and for each interval given the background markers (TR^2^) to determine the phenotypic variance explained by a single QTL. LOD values were calculated using the formula LOD = 0.2172 * LRT. Multiple linear regression for each chromosomal position was applied at the 5% significance level to obtain the cofactors used in the analysis. Threshold values were identified for each trait based on 1000 permutations and represented by graphs using Excel 2010.

### Statistical analysis

Analysis of variance (ANOVA) and the Generalized Linear Models (GLM) procedure were to assess the performances of the RILs and of each trait evaluated. All data and statistical analyses were done using the software SAS v.8.2 (SAS Institute, Cary, NC, USA). A value of p < 0.05 indicated significance.

## Results

### Marker characteristics

Among the 594 microsatellite markers screened in the parents, 150 (25%) were polymorphic for the population and 80 SSRs (53%) were mapped. SNP profiling produced 288 polymorphic markers, 251 of which were used in genetic mapping. The linkage map was constructed with a total of 331 markers that segregated among the population and covered all 11 bean chromosomes, with a total length of 1515.2 cM. All markers were distributed across the bean genome, with an average density of 4.5 cM. The size of the chromosomes ranged from 63.1 cM (Pv 10) to 221.2 cM (Pv 1, Table 1). The highest saturation was found for Pv 3, with 40 markers, including 30 SNPs and 10 SSRs.

**Table 1:** Distribution of SSRs and SNPs mapped in the 11 chromosomes of the common bean genetic map from the AND 277 x SEA 5 population.

Linkage group (Pv)	SSR	SNP	No. of linkage loci	Linkage length (cM)	Average distance (cM)
1	10	26	36	221.2	6.1
2	11	21	32	161.4	5
3	10	30	40	159.4	3.9
4	5	18	23	128.4	5.5
5	5	22	27	147.2	5.4
6	8	19	27	148.3	5.4
7	8	22	30	179.6	5.9
8	7	22	29	86.7	2.9
9	7	17	24	112.4	4.6
10	5	23	28	63.1	2.2
11	4	31	35	107.5	3
**Total**	80	251	331	1515.2	4.5

cM – CentiMorgan, SNP – single nucleotide polymorphism, SSR – simple sequence repeat.

### Field conditions

**Figure 1:**
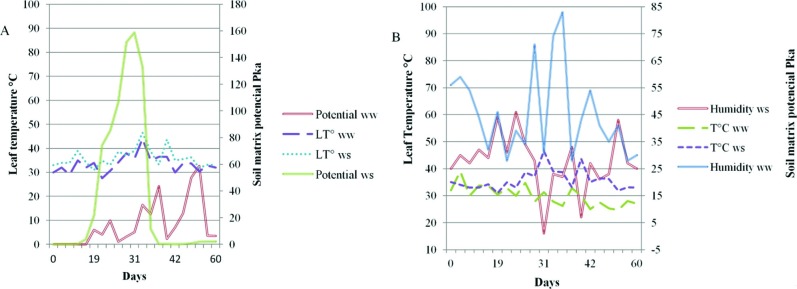
Environmental parameters and soil matrix potential measured every two days during the first 60 days after planting. (A) Leaf temperature and soil water tension, (B) Greenhouse humidity and temperature.

The well-watered greenhouse or control treatment was kept at 80% field capacity throughout the experiment, with an average temperature of 34 °C and 52% relative humidity. The water-stressed greenhouse was under terminal stress conditions in the vegetative phase (V3/V4) and had an average temperature of 36.4 °C and relative humidity of 42.4%. Humidity was lower in the stressed greenhouse and leaf temperatures showed almost the same pattern, with higher temperatures in the stressed greenhouse (Figure 1).

Descriptive statistics and analysis of variance of the morphological response patterns related to drought tolerance detected significant differences among the parents and RILs for most of the traits (Tables 2 and S1). In the well-watered greenhouse, the parental lines, SEA 5 and AND 277, differed in leaf dry biomass, leaf temperature, days to flowering, number of pods, number of seeds per pod, yield, number of seeds, seed weight and pod weight. Among the RILs, all the traits were significant and showed a normal distribution (Figure S1). The two parents were similar in terms of chlorophyll, leaf area, leaf fresh biomass, stem fresh biomass and stem dry biomass. Heritability was lower for pod weight (0.28) and higher for leaf fresh biomass (0.93).

**Table 2:** Analyses of variance for quantitative traits for AND 277, SEA 5 and recombinant inbred lines of the AS population evaluated in a greenhouse under irrigated and non-irrigated (water-stressed) conditions.

Trait	Irrigated					Non-irrigated				
	Parents		Diff	Mean RILs	h^2^_g_	Parents		Diff	Mean RILs	h^2^_g_
	SEA 5	AND 277				SEA 5	AND 277			
Chlorophyll	42.46	43	ns	41.69*	0.54	23.85	26.96	ns	26.81*	0.71
Leaf area	2098.7	2315.3	ns	1402.59*	0.75	390.25	149	*	303.96*	0.87
Fresh leaf biomass	32.83	37.5	ns	26.59*	0.93	4.75	1.83	*	3.17 ns	0.21
Stem biomass fresh	23.83	24.66	ns	17.07*	0.89	5.87	4.16	ns	4.92*	0.56
Dry leaf biomass	4	6.83	*	3.47*	0.64	1.87	1	*	1.01 ns	0.12
Dry stem biomass	3.33	4.33	ns	2.04*	0.36	1.25	1.5	ns	1.18*	0.39
Leaf temperature	23	30.33	*	28.25*	0.61	33.33	35.5	*	34.63*	0.44
Days to flowering	31	37.75	*	36.77*	0.92	38	36	*	36.37*	0.94
Number of pods	13	5.25	*	10.56*	0.49	13	5	*	14.53*	0.53
Number of seeds/pod	4.23	2.32	*	2.8*	0.64	4.5	2.7	*	2.75*	0.73
Yield (g/plant)	9.46	4.6	*	6.67*	0.9	11.99	3.52	*	7.74*	0.95
Number of seeds	53.33	12.25	*	27.79*	0.35	48	10.5	*	37.65*	0.46
Seed weight (g/100 seeds)	21.88	38.68	*	22.02*	0.84	23.18	12.47	*	23.89*	0.73
Pod weight	4.14	1.14	*	2.28*	0.28	4.33	2.12	*	3.87*	0.68

Diff – difference between parents, h^2^_g_ – heritability.

* p < 0.05; ns – not significant.

In contrast, in the water-stressed greenhouse, the parental lines differed in leaf area, leaf fresh biomass, leaf biomass, dry weight, leaf temperature, days to flowering, number of pods, number of seeds per pod, yield, number of seeds, seed weight and pod weight. Among the RILs, leaf fresh biomass and leaf biomass dry weight were not significant traits. Heritability was lower for leaf biomass dry weight (0.12) and higher for yield (0.95).

In the well-watered treatment, the mean yield of all the genotypes was 2.84 g/plant, and 1.97 g/plant in the treatment under drought stress. This result showed a 30% reduction in grain yield due to drought, calculated using the drought intensity index. Parental means were significantly different, except for leaf fresh and dry biomass.

### QTL mapping

**Figure 2:**
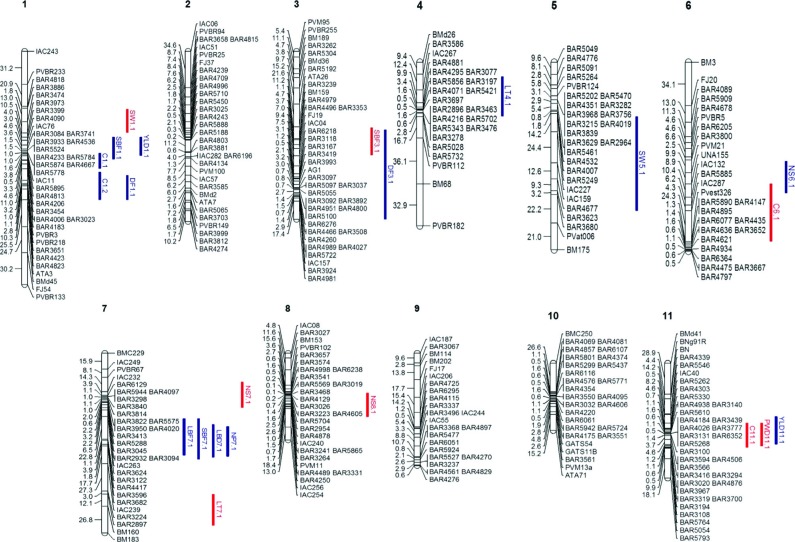
Common bean linkage map constructed using the AND 277 x SEA 5 RIL population. The positions of the QTLs for drought (blue) and irrigated treatments (red) are shown. Chromosomes were assigned based on the *P. vulgaris* L. genome (http://www.phytozome.net/).

Of 22 QTLs identified in the experiment, eight were under drought conditions and 12 under irrigation conditions (Tables 3 and 4, Figure 2). Dry pod weight was detected only under drought treatment and explained 17% of the phenotypic variance with a negative allele for the QTL, indicating that SEA 5 contributed to this trait, with an LOD of 3.48 and a BAR3100 marker located within the QTL (Table 4). The QTLs were detected in both conditions but appeared on different chromosomes (Figure 2). The greatest amount of phenotypic variance associated with drought tolerance was detected for the chlorophyll QTL, with a coefficient of determination (R^2^) of 32.8%. However, this trait was also detected in the irrigated condition and explained 32.1% of the phenotypic variance. For drought treatment, stem fresh biomass, seed weight and number of seeds (g/100 seeds) showed the greatest effects with R^2^ (18%, 17% and 15%, respectively). These QTLs were found linked to the BM159 marker in Pv 3, the BAR3474 marker in Pv 1 and the BAR3045 marker in Pv 7, with a contribution from the SEA 5 allele. All the QTLs detected under drought treatment showed a contribution from the SEA 5 parental allele, except for one chlorophyll QTL identified in Pv 11 and one QTL for leaf temperature identified in Pv 7. Fresh and dry biomass had a positive allele from AND 277 under irrigated treatment, whereas leaf temperature had a positive allele from AND 277 under both treatments. The number of pods and days to flowering under the irrigated treatment and the number of seeds and seed weight under both treatments had a negative allele from SEA 5. Positive and negative alleles contributed to yield in the irrigated treatment. Most of the QTL identified a contribution from SEA 5 (13) rather than from AND 277 (9).

**Table 3:** Identification of quantitative trait loci for chlorophyll, fresh leaf biomass, fresh stem biomass, dry leaf biomass, leaf temperature, number of pods, number of seeds, seed weight, days to flowering, dry pod weight and yield for the AS population.

Trait	QTL	Treatment	Pv	Marker interval	Marker	LOD score	LOD threshold	Additive effect	R^2^ (%)
Chlorophyll	C1.1^AS^	Irrigated	1	114.7-140.7	BAR3651	9.8	3.8	0.36	32.88
Chlorophyll	C1.2^AS^	Irrigated	1	96.83-111.08	BAR3454	3.5	3.8	-0.21	11.23
Chlorophyll	C6.1^AS^	Drought	6	95.54-140.45	BAR5885	10.8	3.1	-3.87	32.12
Chlorophyll	C11.1^AS^	Drought	11	56.23-78.68	BAR3594	4.3	3.1	2.14	11.42
Fresh leaf biomass	LBF7.1^AS^	Irrigated	7	65.3-101.92	BAR3122	4.1	3.1	0.13	13.66
Fresh stem biomass	SBF1.1^AS^	Irrigated	1	81.43-102.44	IAC11	3.3	3	0.07	10.26
Fresh stem biomass	SBF7.1^AS^	Irrigated	7	65.3-105.92	BAR4417	3.8	3	0.08	13.2
Fresh stem biomass	SBF3.1^AS^	Drought	3	72.55-98.25	BM159	4.1	2.9	-0.11	18.26
Dry leaf biomass	LBD7.1^AS^	Irrigated	7	71.3-103.92	BAR4417	3	3	0.04	11.29
Leaf temperature	LT4.1^AS^	Irrigated	4	11.41-42.23	BAR4881	3.4	3	0.61	12.38
Leaf temperature	LT7.1^AS^	Drought	7	140.9-171.98	BAR2897	3	3	0.57	14
Number of pods	NP7.1^AS^	Irrigated	7	73.3-102.92	BAR3682	3.2	3.2	-0.06	12.63
Number of seeds	NS6.1^AS^	Irrigated	6	77.71-102.54	PVM21	3.3	3.1	-0.38	12.87
Number of seeds	NS7.1^AS^	Drought	7	29-54.34	BAR3045	4.1	2.9	-0.52	15.51
Number of seeds	NS8.1^AS^	Drought	8	39.86-63.42	BAR4250	4.1	2.9	-0.04	0.09
Seed weight (g/100 seeds)	SW5.1^AS^	Irrigated	5	42.67-116.38	BAR4677	3.9	3	-0.29	14.41
Seed weight (g/100 seeds)	SW1.1^AS^	Drought	1	54.88-76.87	BAR3474	4.4	3	-0.12	17.32
Days to flowering	DF1.1^AS^	Irrigated	1	117.7-140.76	BAR4823	3.2	3.1	-0.01	10.8
Days to flowering	DF3.1^AS^	Irrigated	3	74.55-159.28	BAR3097	5.1	3.1	-0.02	14.37
Dry weight pod	PWD11.1^AS^	Drought	11	56.23-77.52	BAR3100	3.5	3.1	-0.15	14.19
Yield (g/plant)	YLD1.1^AS^	Irrigated	1	81.43-98.87	BAR3084	3.2	3.1	0.19	10.08
Yield (g/plant)	YLD11.1^AS^	Irrigated	11	50.03-75.83	BAR4938	3.5	3.1	-0.19	11.86

R^2^ – coefficient of determination %

**Table 4:** Identification of quantitative trait loci for significant drought tolerance in the AS population, their marker interval, marker located nearest to the QTL peak and its distance from the peak (in cM).

Trait	Linkage group (Pv)	Interval (cM)	Marker (distance to the peak)	R^2^ (%)	Additive effect
Chlorophyll	6	95.54–140.45	BAR5885 (0 cM)	32.1	-3.87
Chlorophyll	11	56.23–78.68	BAR3594 (0 cM)	11.4	2.14
Fresh stem biomass	3	72.55–98.25	BM 159 (2 cM)	18.3	-0.11
Leaf temperature	7	140.9–171.98	BAR2897 (6 cM)	14	0.57
Number of seeds	7	29–54.34	BAR3045 (3 cM)	15.5	-0.52
Number of seeds	8	39.86–63.42	BAR4250 (8 cM)	0.09	-0.04
Seed weight (g/100 seeds)	1	54.88–76.87	BAR3474 (0 cM)	17.3	-0.12
Dry weight pod	11	56.23–77.52	BAR3100 (0 cM)	14.2	-0.15

R^2^ – coefficient of determination %

## Discussion

When compared to other maps (Campos *et al.*, 2011; Blair *et al.*, 2012; Oblessuc *et al.*, 2014) the position of the markers remained the same on the 11 chromosomes, thus confirming the robustness and reliability of the genetic map generated by this study. The markers were placed on all 11 chromosomes and covered the whole genome, thereby allowing identification of the QTLs under two irrigation systems. The average genetic distance between markers was 4.5 cM and therefore provides a dense map ideal for QTL analysis.

Exposure of the plants to drought stress substantially decreased the leaf water potential, relative water content and transpiration rate, with a concomitant increase in leaf temperature (Siddique *et al.*, 2001). SEA 5 had cooler leaves than AND 277, apparently by reducing the leaf temperature in drought conditions (Table 2).

Three types of drought stress are commonly recognized, with two types of water supply (irrigated for non-stress and rain-fed for drought stress) being used to assess the effects of the intensity and duration of drought on crop growth and seed yield in genetically fixed materials (Blair *et al.*, 2012; Sabadin *et al.*, 2012). In this study, terminal drought stress was chosen because it affects over 60% of dry bean production worldwide (White and Singh, 1991), with the most affected areas in Latin America being northeastern Brazil and Central America. Although terminal drought stress is one of the most severe types of drought, the results of this experiment found a 30% reduction in grain yield and, surprisingly, even with the large reduction, some of the RILs had higher yields under terminal drought stressed conditions. These results corroborated those of Acosta-Diaz *et al.* (2004) and may be explained by the observation that the drought allele (an allele for a favorable environment) was accompanied by a neutral allele for the other environment. This implies that yield under drought conditions and yield under well-watered conditions are not mutually exclusive and can be combined (Beebe *et al.*, 2011). Analysis of variance of the quantitative traits showed that the SEA 5 parental line was significantly superior for almost all the traits measured under water stress conditions, except for leaf temperature. Under normal conditions, the days to flowering trait in SEA was greatly inferior to that of AND 277 under well-watered conditions and was greatly delayed under stress conditions (but was significant in both cases). The performance of SEA 5 with regard to the number of seeds/pod, yield, seed weight and pod weight was better under drought (stress) compared to well-watered conditions, whereas the number of pods was unaffected.

Eight QTLs for drought conditions were identified and showed different levels of genetic variability; these QTLs were located on chromosomes 1, 3, 6, 7, 8 and 11. All the QTLs identified under drought conditions had the SEA 5 allele, except for the QTL for leaf temperature (LT7.1^AS^ – leaf temperature). Fourteen QTLs were identified in the irrigated environment, with R^2^ values ranging from 10% to 33%, and were located on chromosomes 1, 3, 4, 5, 6, 7 and 11. QTLs were identified in all chromosomes except for chromosomes 2, 9 and 10. Overlapping QTLs were identified in chromosomes 1, 3, 6, 7 and 11. Blair *et al.* (2012) also found some overlapping QTLs and suggested that pleiotropic genes controlled two or more traits. Mukeshimana *et al.* (2014) found that correlated variables such as phenology, yield and yield components, co-localized on the same chromosome and that the yield QTL occurred mainly on Pv03 and Pv09. In the present study, leaf fresh biomass, stem fresh biomass, leaf biomass dry weight and the number of pods clustered together in Pv 7 under well-watered conditions. Although LBD7.1^AS^ (dry leaf biomass) showed a higher marker interval, it showed the same marker for the QTL peak (BAR4417) as SBF7.1^AS^ (fresh stem biomass).

QTLs for chlorophyll, stem fresh biomass, leaf temperature, number of seeds and seed weight were identified in both treatments. For days to flowering, leaf fresh biomass, leaf biomass dry weight, number of pods and yield, QTLs were detected only in the irrigated treatment. For pod dry weight, a QTL was identified under drought conditions. The seed weight trait was important given that seed filling is inhibited under drought stress, so large seeds may indicate tolerance to drought and lead to higher yields (Ramírez-Vallejo and Kelly, 1998). Furthermore, the allele for seed size under drought and irrigated conditions came from the drought-tolerant parent, SEA 5. Two QTLs were found for seed weight, one in Pv 1 and the other in Pv 5. Blair *et al.* (2012) also found QTLs for seed weight in Pv 5 and Broughton *et al.* (2003) found QTLs in Pv 1. QTLs for days to flowering were found in Pv 1 and 3, while Broughton *et al.* (2003) located them in Pv 1 and 8. According to Mukeshimana *et al.* (2014), Pv03 also seems to be related to the seed weight QTL.

The nature of drought and its interaction with multiple environmental factors make the validation of QTLs much more complex. Schneider *et al.* (1997) studied the genetics of drought resistance using QTLs detected with RAPD markers. Four markers in one population and five in a second RIL population were reported to be important for drought resistance. Beebe *et al.* (2006) reported the identification of markers for QTLs under drought and irrigated conditions in a RIL population derived from the SEA 5 x MD 23-24 cross; one QTL was common to two drought seasons, one was specific to each of two seasons, and some were common to unstressed environments. Blair *et al.* (2012) identified several QTLs in a BAT477 x DOR364 RIL, most of them being for seed weight followed by yield per day, yield *per se*, days to flowering and days to maturity. However, these authors noted that fewer QTLs were detected in the first year because of differences in the severity of drought stress and in the experimental conditions form year to year (terminal *vs.* intermittent drought).

In terms of breeding for drought tolerance, BAT 477 has been widely used to improve various classes of common beans (Terán and Singh, 2002). SEA 5 is an advanced line derived from BAT 477 that proved to have a superior background in terms of donor alleles favorable to QTLs associated with drought tolerance, as shown here. Mukeshimana *et al.* (2014) reported that the only QTL associated with yield under drought stress on Pv09 was contributed by the SEA 5 parent in combined environments, indicating the importance of SEA 5 alleles in maintaining yield under drought stress. Gonçalves *et al.* (2015) studied the combining ability under drought stress in common bean cultivars recommended for breeding programs aimed at drought tolerance, with grain yield as the parameter. Common beans of the Durango race, such as SEA 5 from the semi-arid highlands of Mexico, have been reported to have the highest levels of drought resistance (Terán and Singh, 2002). Thus, combining the germplasms of Durango and Mesoamerica races, such as SEA 5 x AND 277, may provide a consistent source of improved drought resistance for tropical environments (Mukeshimana *et al.*, 2014).

Since the nature of drought and its interaction with multiple environmental factors makes QTL validation much more complex the challenge will be to test combined populations across broad classes of environments to determine which QTLs are stable. The complexity of this task will assist in rationally establishing an effective approach for marker-assisted selection (MAS). Beebe *et al.* (2013) suggested testing a subsample of 30-40 phenotypically extreme segregant RILs in a smaller trial over multiple sites for the sole purpose of validating the QTLs. Schneider *et al.* (1997) validated markers using a small set of selected RILs. Multiple environment trials should be done and QTL mapping confirmed in order to estimate genotype x environment (G x E) interactions.

The results of this study indicate that SEA 5 and AND 277 parents had contrasting sensitivities to drought tolerance, with SEA 5 having a superior background in terms of donor alleles favorable to QTLs associated with drought tolerance. The SEA 5 genotype was superior for drought tolerance for traits such as leaf area, pod dry weight and yield. Genotyping with SSRs and SNPs showed a high level of polymorphism in the AS population and a high level of map saturation. Among QTLs associated with water deficit, 75% had a contribution from the SEA 5 genitor. For QTLs relevant to the cultivation of common beans, those related to leaf area, fresh mass and pod dry weight were the most important ones. Leaf foliar temperature was not a useful trait for future studies of QTLs associated with drought tolerance.

Drought tolerance is a complex quantitative trait controlled by many minor QTLs. This study confirmed that molecular markers are powerful tools for a better understanding of the molecular basis of drought tolerance in the common bean and, once validated, can be used in molecular breeding.

## Supplementary material

The following online, material is available for this article:

Figure S1 - Distribution of quantitative traits across the RILs.

Table S1 - Descriptive statistics for the quantitative traits evaluated in the AND 277 x SEA 5 population.
