# Supplementary material for: Mapping QTLs for drought tolerance in a SEA 5 x AND 277 common bean cross with SSRs and SNP markers
Source: Genet Mol Biol. 2017 Oct 23;40(4):813–23. doi: 10.1590/1678-4685-GMB-2016-0222 (PMC5738610; doi:10.1590/1678-4685-GMB-2016-0222)
Supplement: Supplementary file 1 [file 1415-4757-gmb-1678-4685-GMB-2016-0222-Suppl02.pdf]

Supplementary Material to “Mapping QTLs for drought tolerance in a SEA 5 x AND 277 common bean cross with SSRs and SNP markers”

Table S1 - Descriptive statistics for the quantitative traits evaluated in the AND 277 x SEA 5 population.

Trait	Skewness	Kurtosis	Shapiro-wilk	*p* value
CI[a]	0.2521	23.155	0.9743	<0.0001*
CNI[a]	0.3265227	0.5741205	0.987999	0.0056*
LAI[b]	32.192.017	5.174.894	0.668798	<0.0001*
LANI[b]	0.2524645	12.333.302	0.981124	0.0003*
FWLI[c]	0.6048856	33.423.528	0.958267	<0.0001*
FWLNI[c]	0.3104951	0.8344974	0.985677	0.0045*
FWSI[d]	0.5038602	20.778.767	0.968003	<0.0001*
FWSNI[d]	0.5471411	0.3567925	0.980573	0.0002*
DLI[e]	0.2278624	21.416.397	0.976136	<0.0001*
DLNI[e]	0.2124753	-0.434425	0.989543	0.0216[ns]
DMSI[f]	0.385813	12.699.492	0.984967	0.0021*
DMSNI[f]	0.0430564	-0.292912	0.993814	0.1802[ns]
TII[g]	0.0003301	-0.602149	0.989967	0.0281[ns]
TINI[g]	0.162045	-0.248152	0.994755	0.2955[ns]
NPI[h]	0.4427387	32.771.693	0.962555	<0.0001*
NPNI[h]	0.5490551	17.796.124	0.974562	0.0001*
SPI[i]	12.567.547	17.002.439	0.865663	<0.0001*
SPNI[i]	0.0109032	25.221.149	0.961073	<0.0001*
NSI[j]	0.8686761	54.055.397	0.950637	<0.0001*
NSNI[j]	0.5657035	13.434.045	0.978579	0.0005*
WSI[k]	10.914.729	60.948.002	0.901571	<0.0001*
WSNI[k]	0.1329638	17.375.184	0.830788	<0.0001*
FI[l]	-1.014.628	46.333.071	0.879631	<0.0001*
FNI[l]	-171.401	1.176.586	0.645045	<0.0001*
DWPI[m]	0.4873849	1.977.694	0.9745	<0.0001*
DWPNI[m]	0.4259785	2.526.472	0.950539	<0.0001*
GYI[n]	25.860.714	20.334.402	0.810543	<0.0001*
GYNI[n]	-0.224504	0.483523	0.989632	0.0504[ns]

[a]CI/CNI – Chlorophyll Irrigated / Non-irrigated
[b]LAI/LANI – Leaf area irrigated / Non-irrigated
[c]FWLI/FWLNI – Fresh weight of leaf Irrigated / Non-irrigated
[d]FWSI/FWSNI – Fresh weight of stem Irrigated / Non-irrigated
[e]DLI/DLNI – Dry leaf Irrigated / Non-irrigated
[f]DMSI/DMSNI: Dry mass of stem Irrigated / Non-irrigated
[g]TII/TINI – Leaf temperature Irrigated / Non-irrigated
[h]NPI/NPNI – Number of pods irrigated / Non-irrigated
[i]SPI/SPNI – Number of seeds per pod irrigated / Non-irrigated
[j]NSI/NSNI – Number of seeds irrigated / Non-irrigated
[k]WSI/WSNI – Weight of 100 seeds Irrigated / Non-irrigated
[l]FI/FNI – Days to flowering Irrigated / Non-irrigated
[m]DWPI/DWPNI – Dry weight of pods Irrigated / Non-irrigated
[n]GYI/GYNI – Grain yield Irrigated / Non-irrigated
*p<0.01; ns – not significant
