# Supplementary material for: Mapping QTLs for drought tolerance in a SEA 5 x AND 277 common bean cross with SSRs and SNP markers
Source: Genet Mol Biol. 2017 Oct 23;40(4):813–23. doi: 10.1590/1678-4685-GMB-2016-0222 (PMC5738610; doi:10.1590/1678-4685-GMB-2016-0222)

Supplementary Material to "Mapping QTLs for drought tolerance in a SEA 5 x AND 277 common bean cross with SSRs and SNP markers"

Figure S1 - Distribution of quantitative traits across the RILs.

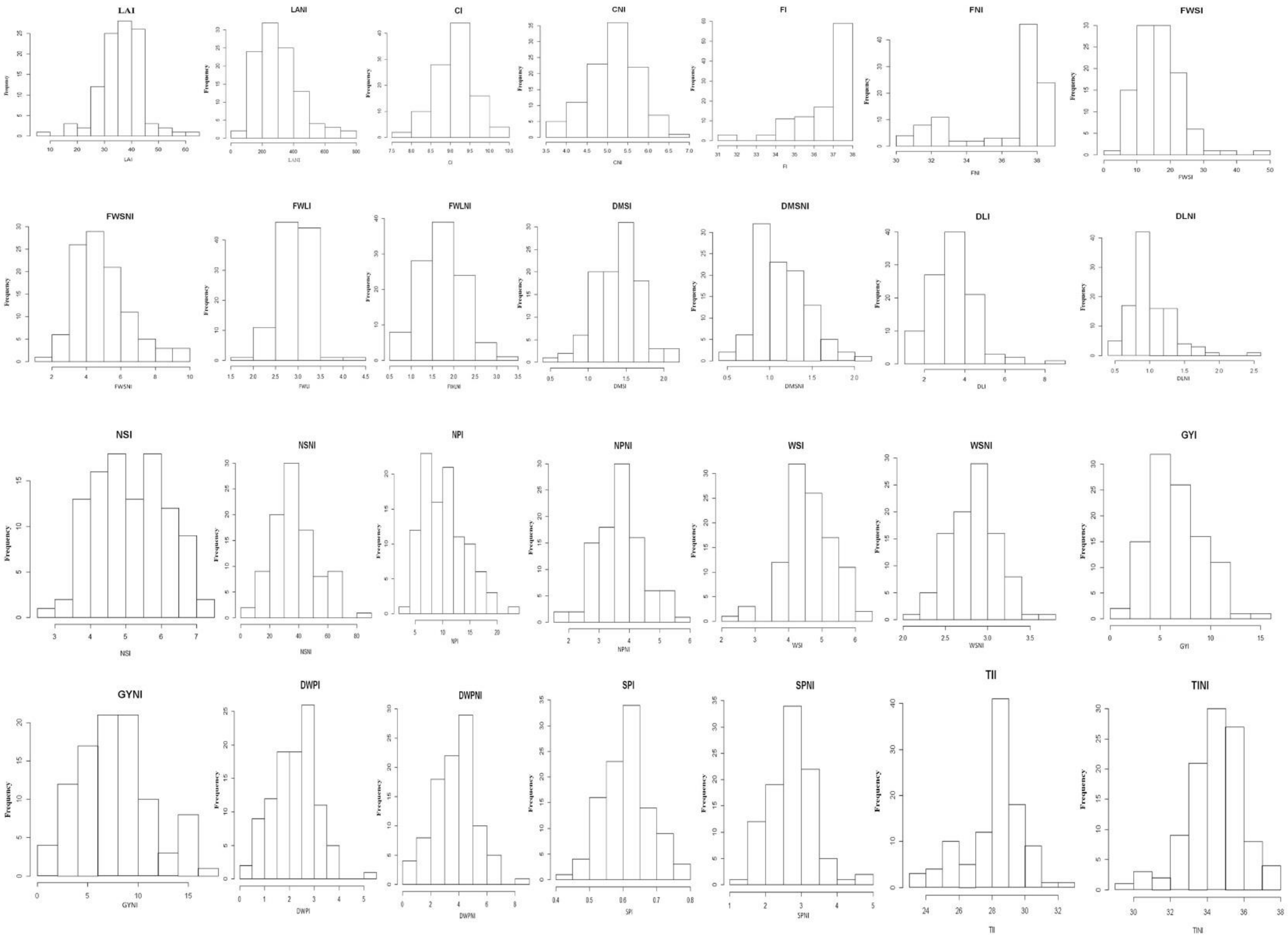

Supplement: Supplementary file 2 [file 1415-4757-gmb-1678-4685-GMB-2016-0222-Suppl01.pdf]
